# Using Microfluidic Hepatic Spheroid Cultures to Assess Liver Toxicity of T-2 Mycotoxin

**DOI:** 10.3390/cells13110900

**Published:** 2024-05-24

**Authors:** Mercedes Taroncher, Alan M. Gonzalez-Suarez, Kihak Gwon, Samuel Romero, Angel D. Reyes-Figueroa, Yelko Rodríguez-Carrasco, María-José Ruiz, Gulnaz Stybayeva, Alexander Revzin, Jose M. de Hoyos-Vega

**Affiliations:** 1 Department of Physiology and Biomedical Engineering, Mayo Clinic, Rochester, MN 55901, USA; mercedes.taroncher@uv.es (M.T.); gonzalezsuarez.alan@mayo.edu (A.M.G.-S.); stybayeva.gulnaz@mayo.edu (G.S.); 2 Research Group in Alternative Methods for Determining Toxics Effects and Risk Assessment of Contaminants and Mixtures (RiskTox), Laboratory of Food Chemistry and Toxicology, Faculty of Pharmacy, University of Valencia, Av. Vicent Andrés Estellés s/n, 46100 Valencia, Spain; yelko.rodriguez@uv.es (Y.R.-C.); m.jose.ruiz@uv.es (M.-J.R.); 3 Centro de Investigación en Matemáticas Unidad Monterrey, Apodaca 66628, NL, Mexicoangel.reyes@cimat.mx (A.D.R.-F.); 4 Consejo Nacional de Humanidades, Ciencias y Tecnologías, Ciudad de Mexico 03940, Mexico

**Keywords:** T-2, HepG2 cells, microfluidic devices, spheroids, cytotoxicity

## Abstract

The *Fusarium* fungi is found in cereals and feedstuffs and may produce mycotoxins, which are secondary metabolites, such as the T-2 toxin (T-2). In this work, we explored the hepatotoxicity of T-2 using microfluidic 3D hepatic cultures. The objectives were: (i) exploring the benefits of microfluidic 3D cultures compared to conventional 3D cultures available commercially (Aggrewell plates), (ii) establishing 3D co-cultures of hepatic cells (HepG2) and stellate cells (LX2) and assessing T-2 exposure in this model, (iii) characterizing the induction of metabolizing enzymes, and (iv) evaluating inflammatory markers upon T-2 exposure in microfluidic hepatic cultures. Our results demonstrated that, in comparison to commercial (large-volume) 3D cultures, spheroids formed faster and were more functional in microfluidic devices. The viability and hepatic function decreased with increasing T-2 concentrations in both monoculture and co-cultures. The RT-PCR analysis revealed that exposure to T-2 upregulates the expression of multiple Phase I and Phase II hepatic enzymes. In addition, several pro- and anti-inflammatory proteins were increased in co-cultures after exposure to T-2.

## 1. Introduction

Mycotoxins are secondary metabolites of low molecular weight produced by filamentous fungi that are often present in food. They may cause adverse effects in the short and long term for consumers depending on the concentrations present in aliments and the exposure time [1]. Mycotoxins are present worldwide in cereals—notably wheat, barley, maize, oats, rye, and rice—and animal feed [2]. Mycotoxins represent one of the most important categories of natural toxins relative to human and animal health. Mycotoxins produce significant economic losses around the world because the value of the crop is reduced when high levels of mycotoxins are detected in the grain [2]. According to the Food and Agriculture Organization (FAO), more than 300 different mycotoxins have been identified in food commodities and animal feed [3]. Among these, the T-2 toxin (T-2) is found in several cereals, predominantly oats [1]. The T-2 is a type A trichothecene (tetracyclic sesquiterpenoid compound characterized by a 12,13-epoxy group and a functional carbonyl group at C-8) produced by several species of *Fusarium* fungi [4]. Some of the described mechanisms of action of this toxin are inhibition of protein synthesis, activation of the mitogen-activated protein kinase (MAPK), apoptosis in various cell types, and inhibition of DNA and RNA synthesis [5]. Studies revealed that T-2 can induce apoptosis of hematopoietic progenitors, blocking the renewal of blood cells in the bone marrow, which leads to a decrease in the number of leukocytes and thrombocytes and increases the risk of septicemia and hemorrhage [6]. It has been pointed out that after ingestion of T-2, its detoxification may not always be completed, producing more adverse effects than expected [7]. Therefore, the European Commission requested that the European Food Safety Authority (EFSA) review the information regarding the toxicity of T-2 and the levels of this mycotoxin that produce adverse effects in humans and livestock [7]. This mandate has opened a new debate regarding the importance of the T-2 toxin and the need for a re-evaluation of its mechanisms of action for a better understanding of its toxicokinetics, adverse effects, and present and future risks.

While animal models may be preferred for the study of T-2 toxin, they are complex, making it challenging to study mechanisms; they also present ethical issues and may not accurately recapitulate human physiology [8]. As a result, there have been extensive studies on T-2 cytotoxicity in vitro in a variety of cell lines such as IPEC-J2, HepG2, C28/I2, Caco-2, and HT-29 cells [9,10,11,12]. Given the reported adverse effects of T-2 on the liver [13], there is considerable interest in modeling liver toxicity and the metabolism of T-2 in vitro. HepG2 cells were derived from hepatocellular carcinoma but retain epithelial hepatic phenotype and are used widely as surrogates for liver studies. These cells have been used by us and others previously to assess the toxicity of T-2 [12,14]. However, these past studies utilized 2D cultures. For example, Ruiz’s team recently revealed an antioxidant defense system of HepG2 cells against the oxidative stress induced by T-2 [15,16]. However, compared to 3D (spheroid) cultures, 2D cultures lack appropriate cell–cell and cell–matrix interactions and may not be sufficiently stable to mimic the physiologic response to toxic compounds [17].

Multiple approaches have been reported to promote the organization of hepatocytes and other liver cells into 3D constructs including spheroids [18,19]. For example, 3D cultures of hepatocytes sandwiched between layers of gel (collagen, heparin, or Matrigel) have been shown to enhance and stabilize hepatic function [20,21,22,23]. Another approach is to use 3D plates (e.g., AggreWell plates) to aggregate cells into uniform spheroids in the absence of the exogenous ECM [19]. Each of these approaches has limitations: the use of gel sandwiches may be costly while spheroids in conventional 3D plates may have a limited window of hepatic function and phenotype maintenance [24].

Microfluidic devices have been used extensively for culturing hepatocytes and other cell types in 3D [25,26]. Microfluidic devices may be used to apply physiological forces as well as to conserve the cells and reagents required for testing. Our team has a long-standing interest in using microfluidic devices where cells are cultured under static conditions while confined to small local volumes and receive nutrients from media reservoirs via transport channels. We showed in several publications that such an arrangement results in the local accumulation of secreted signals that act in an autocrine and paracrine manner to enhance the phenotype of cultured cells [27,28,29,30].

Another strategy employed commonly both in standard and microfluidic hepatic cultures is to introduce nonparenchymal cells to stabilize and enhance hepatic function. This enhancement may be attributed to the increase of juxtacrine and paracrine interactions between both cell types [31,32,33]. For example, it has been shown that the co-culture of liver sinusoidal endothelial cells (LSECs), 3T3 fibroblasts, and Kupffer and LX2 cells enhanced the albumin secretion of hepatic cells in standard and microfluidic devices [29,34,35,36].

In this study, we wanted to evaluate whether microfluidic 3D hepatic cultures, either alone or in co-cultures with nonparenchymal cells, were suitable for assessing the toxicity of T-2. To the best of our knowledge, this is the first effort to evaluate T-2 toxicity in 3D hepatic cultures, microfluidic or otherwise.

## 2. Materials and Methods

### 2.1. Reagents

The following reagents were purchased from Sigma Aldrich (Burlington, MA, USA): Pluronic F-127 (P2443), Tween 20 (P9416), and primer sequences of CYP1A1 and CYP2E1. The primer sequences of GAPDH, CYP1A2, CYP3A4, UGT1A1, MDR1, and MDR2 were purchased from Integrated DNA technologies (IDT). The following reagents were purchased from Thermo Fisher scientific (Waltham, MA, USA): Dublecco’s Modified Eagle Medium (DMEM, MT-10-013-CV), penicillin-streptomycin (15140122), Gibco fetal bovine serum (FBS, 10437028), calcein, ethidium homodimer (Live/Dead Viability/Cytotoxicity kit, L3224), Hoechst 33,342, and Vybrant DiD (V22889). Human albumin kit (E110-125) was purchased from Bethyl Laboratories, Inc. (Montgomery, TX, USA). Standard T-2 toxin (MW: 466.6 g/mol, 21259-20-1) was purchased from Cayman Chemical Company (Ann Arbor, MI, USA). Polydimethylsiloxane (PDMS, Sylgard 184 Silicone Elastomer Kit kit, 2065622) and SU-8 2100 were purchased from Ellsworth (Minneapolis, MN, USA) and Kayaku Advanced Materials (Westborough, MA, USA), respectively.

### 2.2. Fabrication of Microfluidic Devices

The microfluidic devices were comprised of two polydimethylsiloxane (PDMS) layers: (1) the top layer consisting of a flow channel with a culture chamber of 100 µm in height, and (2) the bottom layer that contained an array of 84 microwells of 250 × 300 µm (diameter × depth). The top and bottom layer master molds were fabricated using standard photolithography techniques. First, 4 inch silicon wafers (University Wafer, Boston, MA, USA) were cleaned using oxygen plasma. Next, the wafers were spin-coated using SU-8 2100 negative photoresist to create a 100 µm and 300 µm thickness film for the top and bottom master molds, respectively, as recommended by the manufacturer. Following a soft bake step, the design was micropatterned into the resist using a Micro Pattern Generator (µPG 101, Heidelberg, Germany). After a postexposure bake, the mold was developed in SU-8 developer and hard-baked for 2 h at 135 °C. Afterwards, both molds were exposed to chlorotrimethylsilane for 30 min.

Microfluidic devices were fabricated using standard soft lithography. A mixture of 10:1 weight ratio of PDMS base to curing agent was poured onto the master molds. After degassing for 10 min, the mixture was baked at 80 °C for 20 min. Next, the partially cured PDMS was cast out from the master mold and cut, and the inlet and outlet holes were punched at the most distal part of the flow channel. The top and bottom PDMS layers were aligned and irreversibly bonded using oxygen plasma treatment (PDC-001, Harrick Plasma, Ithaca, NY, USA). Media reservoirs were generated using cloning cylinders (10 mm: Fisher Scientific, Waltham, MA, USA) attached with PDMS to the inlet and outlet holes. Prior to cell seeding, all microfluidic devices were incubated with 2% Pluronic F-127 for at least 1 h to avoid interactions between cells and the PDMS surface and inducing cell–cell aggregation. Lastly, the devices were washed with PBS and sterilized with UV-light for 1 h.

### 2.3. HepG2 Cell Cultures

HepG2 cells [HB-8065™, American Type Culture Collection (ATCC), Manassas, VA, USA] were cultured in T-75 culture flasks in DMEM media supplemented with 1% (*v*/*v*) penicillin-streptomycin and 10% FBS at 37 °C with 5% CO_2_. At 80% confluency, cells were passaged into a new T-75 flask or seeded into Aggrewell^TM^400 plates and/or microfluidic devices. For seeding into Aggrewell^TM^400 and microfluidic cultures, we ensured cell viability was at least 90%.

#### 2.3.1. Cell Culture in Standard Large Volume 3D Plates

HepG2 cell spheroids were generated using commercial 24-well Aggrewell^TM^400 plates following the manufacturer’s protocol. While there are other commercial products suitable for cell spheroid cultures (e.g., f- or u-shaped plates), they are designed to culture one spheroid per well and do not mimic a microfluidic device where an array of spheroids is cultured in the same volume of media. Conversely, Aggrewell plate is designed to create a high-density array of spheroids in the same well and therefore represents a better “large volume” comparison to microfluidic spheroid cultures. Within each well there are 1200 v-bottom sub-wells 400 × 400 µm each. Spheroids form within individual sub-wells. Prior to seeding cells, a plate was first treated with 2 mL anti-adherence rinse solution (STEMCELL Technologies Inc., Vancouver, BC, Canada) and then centrifugated at 1300 g for 5 min. Subsequently, the anti-adherence rinse solution was aspirated, and HepG2 cells were seeded at the density of 3.6 × 10^5^ cells per well. The plates were centrifugated at 100× *g* for 3 min to ensure uniform cell distribution within the microwells and then incubated for 5 days at 37 °C with 5% CO_2_ with daily media exchange. To prevent spheroid washout, 50% of the media volume was collected and exchanged during media exchanges. To analyze the morphology of the spheroids, brightfield images were acquired of the wells during the spheroids culture.

#### 2.3.2. Cell Culture in Microfluidic Devices

***HepG2 monoculture***. To generate HepG2 spheroids in microfluidic devices, a total of 20,000 cells were seeded per device. We pipetted 100 µL of cell suspension at 2 × 10^7^ cells/mL into the device inlet while keeping the outlet empty. The difference in hydrostatic pressure between inlet and outlet drove cells into the culture chamber and the microwells. Once the microwells were filled with cells, the leftover cells were removed from the media reservoirs, and the cells outside of the microwells were washed out. Afterwards, 250 µL of media was added into each reservoir. To ensure spheroids formation, 1% Matrigel was added to the media in the first 24 h of culture. Fresh media were exchanged every 24 h. Compact spheroids were formed after 24–48 h of device seeding.

***Co-culture of HepG2 and LX2 cells***. The stellate cell line LX2 (Sigma-Aldrich, SCC064) was cultured in DMEM media, supplemented with 1% (*v*/*v*) penicillin-streptomycin and 2% FBS in a T-75 culture flask at 37 °C with 5% CO_2_. To generate HepG2-LX2 spheroids, we prepared a cell suspension containing 85% HepG2 and 15% LX2. This ratio was chosen based on the proportions of liver cell types in vivo [37]. The HepG2-LX2 cell suspension was seeded into microfluidic devices as previously described in the HepG2 monoculture section. Fresh media were exchanged every 24 h. Compact spheroids were formed after 24–48 h of device seeding. For LX2 identification in the HepG2-LX2 spheroids, LX2 cells were stained with Vybrant DiD dye prior to mixing with HepG2 cells.

### 2.4. T-2 Toxicity in Microfluidic Devices

To determine the effects of T-2 in HepG2 and HepG2-LX2 spheroids, we added different concentrations of the toxin (0, 15, 30, and 60 nM) 3 days after seeding the cells. Then, the cells were treated for 24 h with the toxin by adding T-2 to the culture media with a final concentration of DMSO at 0.1%. Appropriate controls containing the same amount of solvent were included in each experiment. Four culture conditions were tested: (1) HepG2 spheroids without T-2, (2) HepG2 spheroids with T-2, (3) HepG2-LX2 spheroids without T-2, and (4) HepG2-LX2 spheroids with T-2.

### 2.5. Live/Dead Assay and Analysis

An inverted fluorescence microscope (IX-83, Olympus, Tokyo, Japan) with a 10× objective lens was used to carry out a quantitative assay to evaluate the viability of HepG2 and HepG2-LX2 spheroids after 24 h of T-2 exposure. We used Calcein-AM (green) and ethidium homodimer (red) dyes, corresponding to live and dead cells, respectively. The staining solution was prepared in a 15 mL tube by mixing 1 µL calcein and 2 µL ethidium homodimer per 1 mL media. Then, 250 µL of staining solution was added to each culture chamber and incubated for 30 min at 37 °C. After this incubation, we removed the media and washed with 250 µL PBS per device. At least 3 images with 6 spheroids each were acquired for each condition in brightfield and fluorescence channels for calcein (ex/em 494/517 nm) and ethidium homodimer (ex/em 528/617 nm). For each area, a Z-stack was acquired, and then all fluorescence data were compressed to a single image using the “full projection” feature.

Images were analyzed using a custom-made MATLAB (version R2023a, Natick, MA, USA) script to determine the cytotoxicity of T-2 in mono- and co-cultures. Briefly, images were grouped per condition and for each set of images (i.e., BF, green and red fluorescence channels); the green channel image was used to locate the spheroids. A circular area of 300 pixels was drawn surrounding each spheroid. Images were binarized using a consistent threshold per channel across all groups to obtain the area (A_red_ or A_green_) of each fluorescence channel per spheroid. Cytotoxicity was calculated using the following equation: Cytotoxicity% = 100 × A_red_/(A_green_ + A_red_). Six spheroids per condition were analyzed.

### 2.6. Spheroid Formation Image Analysis

#### 2.6.1. Assessing Area, Solidity, and Circularity of Hepatic Spheroids

HepG2 cells were cultured in commercial 3D plates and microfluidic devices as described in Methods Section 2.3. Brightfield images were acquired immediately after cell seeding (0 h) and every 24 h for five days. For both culture conditions, at least five images containing six spheroids each were acquired at every time point (Figure S1A). Acquired images were analyzed using Python (version 3.11.5, Wilmington, DE, USA) and ImageJ (version 1.54d, Bethesda, MD, USA) to quantify and compare three metrics: (1) area of the spheroid, (2) solidity, and (3) circularity (further description of methodology may be found in Supplementary Data). The area of the spheroid is determined by detecting the number of pixels that the spheroid encompasses in the image. Solidity is a metric that indicates how regular (or “smooth”) the surface of the spheroid is, while circularity is an indicator of how similar to a true circle a spheroid is [38,39,40]. Six spheroids per condition were analyzed.

#### 2.6.2. HepG2 and HepG2-LX2 Spheroid Growth Analysis

HepG2 and HepG2-LX2 spheroids were tracked for 4 days, and bright-field images were acquired at days 3 and 4 after seeding, corresponding with the addition of the mycotoxin (day 3) and 24 h after T-2 exposure (day 4). All spheroids in an image were measured using ImageJ to determine the area of spheroids at both days (before mycotoxin exposure and 24 h after treatment). The relative spheroid growth after 24 h of T-2 (day 4) was compared between HepG2 and HepG2-LX2 spheroids. Six spheroids per condition were analyzed.

### 2.7. Analysis of Hepatic Albumin Production by ELISA

Albumin production of hepatocytes in vitro is typically analyzed to determine the hepatic function. Albumin secretion was measured for each culture format by ELISA of conditioned media using a human albumin kit and following the manufacturer’s instructions.

The albumin ELISA was carried out for: (1) HepG2 spheroids cultured in Aggrewell^TM^400 plates and microfluidic devices for 5 days with daily media exchange. The media from each well or microfluidic chamber were collected on days 3, 4, and 5, and the albumin secreted was measured by an ELISA assay. (2) HepG2 or HepG2-LX2 spheroids were cultured only in microfluidic devices for 4 days with daily media exchanges. Spheroids were treated with 15, 30, and 60 nM of T-2 on days 3 and 4. The media collected at these time points were analyzed for albumin secreted in HepG2 spheroid monocultures and HepG2-LX2 spheroid co-cultures. The medium is collected from 3 devices per condition. In each device, there are 84 spheroids. Therefore, the content of albumin producing 252 spheroids in each condition is analyzed.

### 2.8. RT-PCR Analysis of Hepatic Gene Expression

HepG2 or HepG2-LX2 spheroids were cultured in microfluidic devices for 3 days and exposed to T-2 at 0, 15, 30, or 60 nM for 24 h, as described above. Total RNA was extracted from HepG2 spheroids at day 4 (24 h after T-2 exposure) using the High Pure RNA Isolation Kit and dissolved in nuclease-free water. The RNA concentration was quantified using the NanoDrop One Spectrophotometer (Thermo Fisher Scientific).

Complementary DNA (cDNA) synthesis was performed using the Transcriptor First Strand cDNA Synthesis Kit. Gene expression level was quantified using the SYBR master. RT-PCR was performed with 40 cycles. Relative gene expression level was determined through the Ct method using glyceraldehyde 3-phosphate dehydrogenase (GAPDH) as a housekeeping gene. We carried out RT-PCR analysis for some of the most common enzymes of Phase I (CYP1A1, CYP1A2, CYP2E1, and CYP3A4) and Phase II (UGT1A1) metabolism and transporters of Phase III metabolism (MDR1 and MRP2) in the liver. The gene-specific primers used in this study are listed in Table 1. The spheroids of three devices per condition (252 spheroids per condition) were analyzed.

### 2.9. Secretome Analysis

HepG2 and HepG2-LX2 supernatants from spheroids in microfluidic cultures were collected and analyzed for inflammatory markers by Eve Technologies (Calgary, AB, Canada) using 15-plex assay. The medium is collected from three devices per condition after 24 h of T-2 exposure, in each device there are 84 spheroids. Therefore, the content of markers producing 252 spheroids in each condition is analyzed.

### 2.10. Statistical Analysis

One-tailed unpaired *t*-test analysis with *p* < 0.05 was used for statistical analysis of results. The number of replicates in each assay is specified in Material and Methods Section. Data were expressed as mean ± SD. The data were statistically analyzed using GraphPad Prism (ver. 7; GraphPad Software, La Jolla, CA, USA).

## 3. Results and Discussion

### 3.1. Cultivation of Hepatic Spheroids in Microfluidic Devices vs. Standard Large Volume 3D Plates

**Figure 1:**
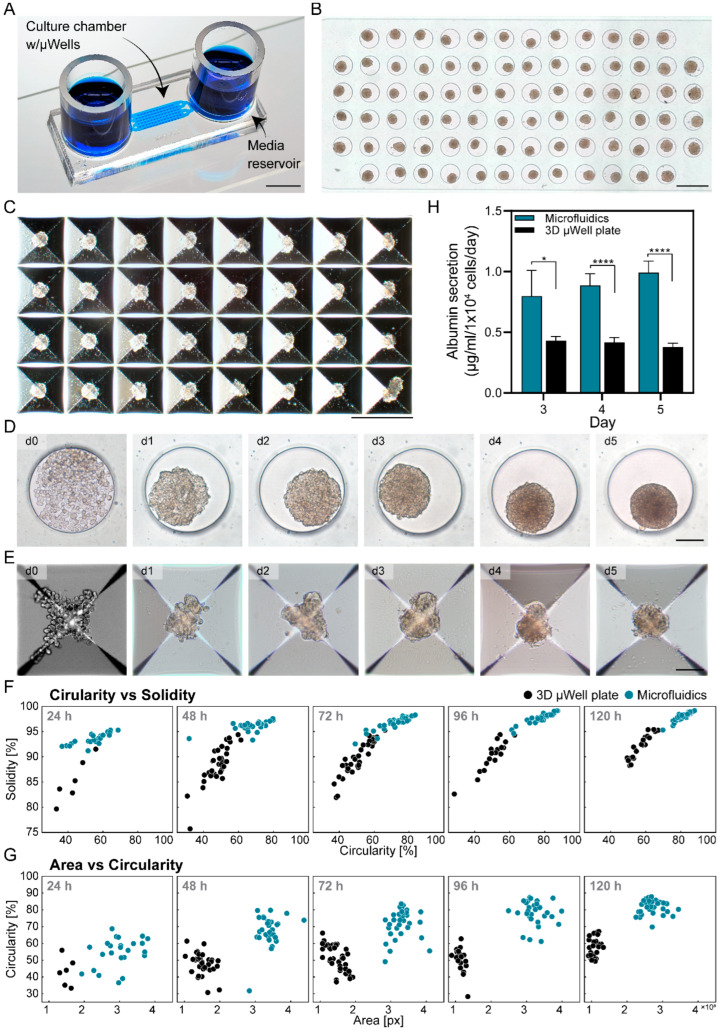
Formation of hepatic spheroids in microfluidic devices and conventional 3D plates. (**A**) Picture of the microfluidic device used for hepatic spheroid cultures; the device contained two reservoirs connected to a cell culture chamber via transport channels. Scale bar: 5 mm. (**B**) Micrograph of the 84 microfluidic microwells with hepatic spheroids at day 5. Scale bar: 500 µm. (**C**) Micrograph of the pyramidal microwell array with HepG2 spheroids after 5 days of culture in conventional (Aggrewell) 3D plates. Scale bar: 500 µm. (**D**) Timelapse images of HepG2 cells aggregating into spheroids after 5 days of culture in a microfluidic device. Scale bar: 100 µm. (**E**) Timelapse images of HepG2 cells aggregating into spheroids after 5 days of culture in a conventional 3D plate. Scale bar: 100 µm. (**F**,**G**) Quantitative comparison of HepG2 spheroid formation measured by (**F**) circularity vs. solidity, and (**G**) area vs. circularity in microfluidic device and conventional 3D plate throughout 5 days of culture. (**H**) Albumin ELISA of HepG2 spheroids in conventional 3D plates and microfluidic devices. Data are expressed as mean ± SD of 3 samples. Statistical significance was determined by one-tailed unpaired *t*-test, * *p*-value < 0.05 and **** *p*-value < 0.0001.

The microfluidic device design was described by us previously [27]. The PDMS device consists of a culture chamber with a height of 100 µm that is connected by two transport channels to the media reservoirs. A culture chamber contains 84 cylindrical wells 350 µm in diameter and 300 µm in depth (See Figure 1A).

We compared spheroid formation in PDMS microwells inside the microfluidic devices vs. a commercial 3D plate (Aggrewell). Both culture systems were seeded with ~250 cells per well and were maintained in the same media. Both culture systems are shown in Figure 1B,C. Importantly, spheroid formation proceeded faster in the microfluidic device compared to the conventional 3D plate. Time course images in Figure 1D,E demonstrate that in a microfluidic device, spheroid formation was nearly complete by day 2, with fully compacted spheroids appearing by day 3. In contrast, in an Aggrewell plate, spheroid formation only began at day 2, with compact spheroids appearing by day 5. Additional image analysis revealed significative differences between Aggrewell and microfluidic cultures in terms of circularity and solidity of spheroids over time (*p* < 0.05 for 24 h and *p* < 0.005 for all other time points). As observed in the scatter plots in Figure 1F, the circularity and solidity of each group of spheroids is well differentiated. For example, with the microfluidic device, spheroids reached solidity values of >90% after 24 h of culture, while spheroids in the Aggrewell plate reached comparable levels after 5 days of culture. Similarly, the circularity of spheroids was 60% after 48 h of culture in a microfluidic device vs. 50% after 120 h of culture in an Aggrewell plate. When comparing the area of the spheroids against their circularity (Figure 1G), it is observed that the spheroids cultured in Aggrewell tended to be smaller and irregular even after 120 h of culture, suggesting that this culture format is not optimal for maintenance and growth of HepG2 spheroids. For microfluidic cultures, we observed that over time, the spheroids become more circular and their area increases, suggesting that the spheroid is becoming more compact and growing, indicating a more suitable culture environment.

To further characterize the difference between culture platforms, the hepatic function of the spheroids was assessed by albumin ELISA. As shown in Figure 1H, the albumin levels in Aggrewell 3D plate were ~2× lower than in the microfluidic devices. Moreover, the production of albumin declined for Aggrewell cultures of spheroids while it increased for microfluidic spheroid cultures.

The enhancement in hepatic cell function observed in the microfluidic devices compared to standard large volume 3D plates can be attributed to several factors including: (1) improved oxygenation in PDMS-based microfluidic devices, and (2) enhanced paracrine signaling due to the small local volume of the microfluidic devices.

The albumin production reported by us is comparable to the albumin values reported by Choi et al. (1.35 µg/1 × 10^4^ cells/day), who cultured primary rat hepatocyte spheroids in microfluidic devices, and significantly better than those reported by Kurosawa et al. (0.014 µg/1 × 10^4^ cells/day), who created hepatocyte monolayer cultures [27,41]. Taken together, our results highlight that microfluidic 3D hepatic cultures were superior to standard large volume 3D culture plates in terms of the dynamics of spheroid formation and albumin production. Therefore, we proceeded to deploy microfluidic cultures for mycotoxin exposure experiments.

### 3.2. Creating Microfluidic 3D Co-Cultures and Assessing Viability upon T-2 Exposure

**Figure 2:**
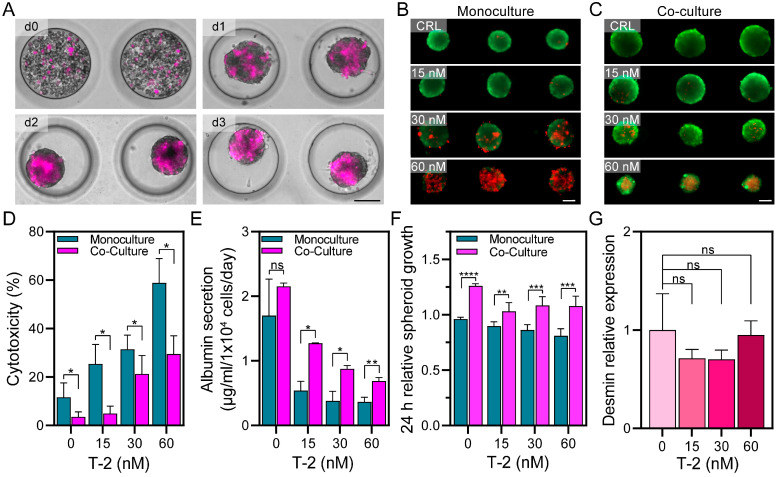
Formation of HepG2-LX2 spheroids and hepatic function assessment in microfluidic cultures. (**A**) Microscopy images of HepG2-LX2 spheroids after 0, 1, 2, and 3 days of culture. LX2 cells were stained with Vybrant DiD (magenta color). Scale bar: 100 µm. (**B**,**C**) Live/dead assay after 24 h of 0, 15, 30, and 60 nM T-2 in HepG2 (**B**) and HepG2-LX2 (**C**) spheroids in microfluidic devices. Live and dead cells took up Calcein-AM (green) and ethidium homodimer (red), respectively. Scale bar: 100 µm. (**D**) Cytotoxicity assessment of HepG2 and HepG2-LX2 spheroids in microfluidic cultures after 24 h exposure to T-2 mycotoxin. (**E**) Albumin ELISA of HepG2 and HepG2-LX2 spheroids in microfluidic cultures after 24 h of T-2 exposure. (**F**) Spheroid growth in monoculture and co-culture after 24 h of T-2 exposure. Changes in spheroid diameter were normalized to day 3 after seeding, corresponding with the addition of the mycotoxin. (**G**) RT-PCR analysis of desmin, a stellate cells marker, gene expression in HepG2-LX2 spheroids after 24 h exposure to varying concentrations of T-2. Data are expressed as mean ± SD of 3 samples. Statistical significance determined by one-tail unpaired *t*-test, * *p*-value < 0.05, ** *p*-value < 0.01, *** *p*-value < 0.001, **** *p*-value < 0.0001.

As noted above, nonparenchymal liver cells have been used in the past to enhance the phenotype and function of hepatocyte cultures. We wanted to test a hypothesis that the presence of nonparenchymal liver cells would further enhance 3D hepatic cultures in microfluidic devices. We employed LX-2, a hepatic stellate cell line, to create co-cultures with HepG2 cells. The proportions of LX-2 cells (15%) and HepG2 cells (85%) were chosen based on the cellular composition of the liver in vivo. LX2 cells were labeled with a cell tracker (Vybrant DiD—magenta color). LX2 cells were aggregated to HepG2 cells to form the spheroids during the first 24 h of culture. As may be appreciated from Figure 2A, after 3 days of cultures, the LX2 cells were homogeneously distributed in the hepatic spheroids. The HepG2 and HepG2-LX2 spheroids were exposed for 24 h to different concentrations of the T-2 mycotoxin (0, 15, 30, and 60 nM), with cell viability analyzed using live/dead staining. As shown in Figure 2B,C, the presence of dead cells increased at higher T-2 concentrations in monocultures. However, this increase was not as pronounced in HepG2-LX2 spheroids. Quantification of live/dead fluorescence (see Figure 2D) confirmed that the monocultures experienced several-fold higher cytotoxicity compared to co-cultures at all concentrations of T-2. Interestingly, the viability of co-culture spheroids at 0 nM T-2 was ~3-fold higher than monoculture spheroids at the same condition. This suggested that stellate cells improved the viability and stability of hepatic cells.

The hepatic function of microfluidic 3D cultures was assessed using albumin ELISA (see Figure 2E). HepG2-LX2 spheroids produced 2.153 µg/mL/1 × 10^4^ cells/day, while HepG2 spheroids produced 1.702 µg/mL/1 × 10^4^ cells/day, showing a tendency of increasing functionality in the co-culture, although the difference is not statistically significant. Albumin production decreased in a concentration-dependent manner in the microfluidic monocultures and co-cultures after 24 h exposure to T-2 (15, 30, and 60 nM). In HepG2 spheroids, the albumin production decreased 68.3% (15 nM), 77.6% (30 nM), and 78.5% (60 nM) after 24 h, with respect to the control (0 nM T-2). On the other hand, microfluidic co-cultures lost 40.8% (15 nM), 59.3% (30 nM), and 68.1% (60 nM) of albumin production when compared to the control. Given that the loss in albumin was more pronounced than the decrease in cell viability (see Figure 2D), we conclude that, in addition to having direct cytotoxic effects, exposure to T-2 also attenuated the hepatic phenotype.

HepG2 cells are capable of proliferation, and we assessed the changes in spheroid diameter for monocultures and co-cultures in the presence of T-2. As shown in Figure 2F, the HepG2-LX2 spheroids were larger than the HepG2 spheroids after 4 days of culture in the absence of T-2. The T-2 exposure caused a greater decline in spheroid growth for monoculture vs. co-culture spheroids.

Are stellate cells affected by exposure to T-2? To address this question, we carried out RT-PCR analysis for desmin, an intermediate filament protein present in stellate cells but not in hepatocytes [42]. This analysis (see Figure 2G) revealed that desmin expression did not change significantly with exposure to T-2, suggesting a minimal cytotoxic effect on these cells. Taken together, our results demonstrate that the microfluidic spheroid co-cultures of hepatic cells and stellate cells exhibited better growth and albumin production and are more resistant to injury compared to 3D hepatic monocultures.

### 3.3. Evaluating the Expression of Hepatic Enzymes in Microfluidic Spheroid Cultures

The liver is the main organ for xenobiotic metabolism. For this reason, in vitro models used to investigate xenobiotic metabolisms often focus on hepatic cells or subcellular fractions, such as microsomes. The metabolism of xenobiotics can be classified in three Phases [43,44,45]. Phase I comprises enzymes responsible for performing the oxidation, hydrolysis, or hydroxylation reactions of xenobiotics. Phase II involves conjugation reactions of the oxidized xenobiotics with glucuronic or sulfuric acid, for example, increasing their hydrophilicity and thus enhancing their secretion through bile and urine. Phase III is formed by transporters, such as multidrug resistance (MDR), that pump conjugated compounds out of the hepatocytes, leading to the limited bioavailability of the substrates. Since the T-2 metabolic pathway is not yet fully understood, several Phase I and II metabolic enzymes and Phase III transporters have been linked to T-2 metabolism [46]. Previously, it was reported that T-2 is rapidly metabolized and may contribute to the overall toxicity of the mycotoxin [47]. It is important to improve our understanding of T-2 metabolism to better understand its mechanism of action in causing liver and systemic toxicity.

**Figure 3:**
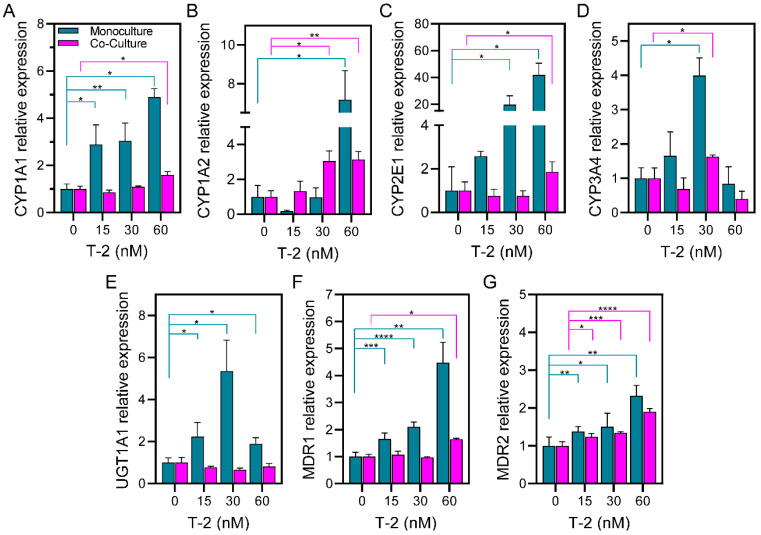
Assessing expression of genes associated with hepatic metabolic activity after exposure to T-2. RT-PCR analysis of phase I (**A**) CYP1A1, (**B**) CYP1A2, (**C**) CYP2E1, and (**D**) CYP3A4, phase II (**E**) UGTA1, and phase III (**F**) MDR1, and (**G**) MDR2 genes associated with the ability to metabolize or transport the mycotoxin. Gene expression results are normalized by 0 h of T-2 exposure condition. Data are expressed as relative expression to the control as mean ± SD of 3 samples. Statistical significance determined by one-tail unpaired *t*-test, * *p*-value < 0.05, ** *p*-value < 0.005, *** *p*-value < 0.001, **** *p*-value < 0.0001.

In the present study, we used RT-PCR to assess the expression of Phase I, II, and III enzymes and transporters for HepG2 and HepG2-LX2 spheroids in microfluidic cultures after 24 h of T-2 exposure. As shown in Figure 3, the expression of CYP1A1 (Figure 3A), CYP1A2 (Figure 3B), CYP2E1 (Figure 3C), CYP3A4 (Figure 3D), UGT1A1 (Figure 3E), MDR1 (Figure 3F), and MDR2 (Figure 3G) was induced by T-2, confirming that this mycotoxin is metabolized and excreted by HepG2 cells. The choice of enzymes was guided by prior reports of the liver metabolism of T-2 and other trichothecenes [48,49,50].

It was important to observe that the microfluidic monocultures of hepatic spheroids responded to 24 h exposure of T-2 by upregulating the expression of all tested enzymes with the exception of CYP3A4 and UGT1A1. Regarding the Phase I metabolism enzymes, the expression levels of CYP1A1, CYP1A2, CYP2E1, and CYP3A4 increased 3.89, 6.17, 40.81, and 2.99-fold, respectively, compared to microfluidic spheroids not exposed to T-2. In terms of Phase II metabolism enzymes, UGT1A1 increased 4.35-fold compared with the control. Lastly, the expression levels of MDR1 and MDR2 were 3.48 and 1.32-fold higher, respectively, compared with their controls. Of all the enzymes tested, CYP2E1 was induced the most (~41 fold compared to control), pointing to an important role played by this cytochrome in T-2 metabolism. An important role of CYP2E1 in T-2 metabolism was previously reported by Lin et al. [51]. Furthermore, CYP2E1 is involved in the metabolism of other mycotoxins produced by *Fusarium* fungi. For example, it is the main cytochrome involved in the metabolism of zearalenone (ZEA) [50].

The expression of metabolizing enzymes in microfluidic 3D co-cultures was interesting and counterintuitive. We did observe the induction of some metabolizing enzymes (e.g., CYP1A1—0.59-fold at 60 nM T-2, CYP1A2—2.14-fold at 60 nM T-2, CYP2E1—0.86-fold at 60 nM T-2). However, the levels of enzyme induction were much lower in co-cultures with stellate cells compared to monocultures of HepG2 cell spheroids, except in the case of MDR2 and CYP3A4, where the decrease of induction is not significant or only significant at 30 nM T-2. This observation is counterintuitive in light of the fact that co-cultures showed better, albeit marginally, production of albumin (see Figure 2). Thus, one could expect similar or better induction of metabolizing enzymes in co-cultures compared to monocultures. While the reasons for the lower induction of metabolizing enzymes in co-cultures remain to be elucidated, there have been literature reports describing stellate cells sequestering mycotoxins in lipid droplets, thus neutralizing their effects in the liver [52,53]. We hypothesize that something similar may be happening in microfluidic co-cultures, and such sequestration of mycotoxin may explain the lower cytotoxicity and better albumin production in co-cultures exposed to T-2 (see Figure 2).

The metabolism of T-2 is complicated because more than 10 metabolites have been identified [51]. According to Lin and colleagues, the CYP450 enzymes that contribute to T-2 metabolism include CYP3A4, CYP2E1, CYP1A2, CYP2C9, CYP2B6, CYP2D6, and CYP2C19, in decreasing order of contribution [51].

Phase III metabolism in HepG2 cells has not been widely studied under T-2 exposure. However, there are studies confirming the importance of this phase in HepG2 cells [54]. In this work, we demonstrate that MDR1 and MDR2 were engaged in T-2 excretion in microfluidic monocultures (Figure 3F,G). Our observations are corroborated by previous studies with intestinal epithelial cells that showed such mycotoxins as deoxynivalenol (DON), nivalenol (NIV), and ochratoxin A (OTA) to be substrates for MDR2 [55,56,57]. In summary, our results demonstrate that microfluidic hepatic spheroids expressed Phase I, II, and III metabolizing enzymes/transporters and that these enzymes/transporters were induced by T-2. Microfluidic hepatic monocultures exhibited much higher enzyme induction compared to co-cultures with stellate cells. Our results highlight that microfluidic spheroid cultures represent an excellent model for investigating mycotoxin effects on liver metabolism. The mechanisms by which stellate cells attenuate the induction of metabolizing enzymes may involve sequestration/neutralization of T-2 but require further investigation.

### 3.4. Assessing Inflammatory Markers in Microfluidic Hepatic Spheroid Cultures

Exposure to T-2 has been shown to affect intestinal barrier function and may lead to pathogen infections, inflammatory bowel diseases (IBD), and mucinous adenocarcinomas [58]. To the best of our knowledge, the inflammatory effects of T-2 have not been explored in liver cultures to date. However, other trichothecenes that also produce gastrointestinal inflammation, such as DON, have been associated with the increased production of inflammatory cytokines (TNF-α and IL.6) in porcine hepatocytes and co-cultures of hepatocytes and Kupffer cells [59,60].

**Figure 4:**
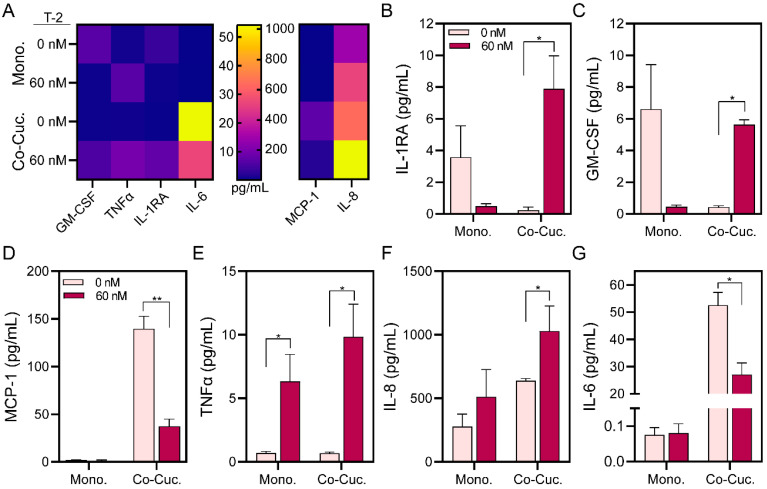
Secretome analysis of microfluidic hepatic spheroid cultures. (**A**) Heatmap of secreted signals, IL-1RA (**B**), GM-CSF (**C**), MCP-1 (**D**), TNF-α (**E**), IL-8 (**F**), and IL-6 (**G**) in monoculture and co-culture spheroids after 24 h of 60 nM T-2 exposure. Data are expressed as mean ± SD of three samples. Statistical significance was determined by one-tail unpaired *t*-test, * *p*-value < 0.05, ** *p*-value < 0.01.

Therefore, in this study, we wanted to evaluate changes in the production of inflammatory cytokines after T-2 exposure in microfluidic hepatic spheroid cultures. Both monoculture and co-culture formats were evaluated. Of the 15 inflammatory biomarkers analyzed using a commercial multiplexed immunoassay panel, we focused on 6 proteins (IL-1RA, GM-CSF, MCP-1, TNF-α, IL-8, and IL-6) that were significantly affected by exposure to 60 nM T-2 (See Figure 4A).

IL-1RA acts as an anti-inflammatory cytokine by binding to and blocking access to the IL-1 receptor without causing downstream signaling [61]. The opposing trends for IL-1RA levels in monocultures and co-cultures in Figure 4A may have several explanations. It is plausible that there are inflammatory processes underway in spheroid monocultures in the absence of mycotoxin and that the higher level of IL-1RA in monocultures may indicate anti-inflammatory response. This hypothesis is supported by the results in Figure 2D that show a much greater loss of viability in monocultures compared to co-cultures in the absence of mycotoxin exposure. The increase in IL-1RA levels in co-cultures after exposure to T-2 may point to a mounting anti-inflammatory response (Figure 4B).

GM-CSF is a proinflammatory and regulatory cytokine associated with the proliferation, differentiation, and chemotaxis of immune cells, epithelial regeneration, and liver fibrosis [62]. Like IL-1RA, GM-CSF may function as a regulatory cytokine during monoculture in the absence of mycotoxin. However, in co-cultures with T-2 it may be involved in a pro-inflammatory response orchestrated by stellate cells (Figure 4C). This could explain the increase of IL-1RA levels in co-cultures with T-2 to counteract the pro-inflammatory response of GM-CSF (see Figure 4A).

MCP-1 is a pleiotropic signal that may be produced by immune cells to promote the migration and infiltration of other immune cells into the injured site but may also be secreted by stellate cells [63]. Given that levels of MCP-1 were negligible in monocultures but robust in co-cultures (Figure 4D), this suggests that stellate cells may be a source of MCP-1 in our cultures [63,64]. The decline of MCP-1 levels in co-cultures exposed to T-2 may be attributed to injury or cell damage.

TNF-α and IL-8 are canonical pro-inflammatory cytokines that may be produced by epithelial, stromal, and immune cells during injury [65]. The production of both cytokines increased in mono- and co-cultures exposed to T-2, suggesting that mycotoxin triggers inflammatory responses in hepatic cultures (Figure 4E,F).

IL-6 was another cytokine expressed at high levels in microfluidic spheroid cultures. It exhibited higher expression in co-cultures than in monocultures, with T-2 exposure causing no change in monocultures but a decrease in co-cultures (see Figure 4G). IL-6 is a complex, pleiotropic molecule known to exhibit either pro- or anti-inflammatory effects depending on the context (type of injury, biological system, etc.). In the liver, IL-6 may be produced by hepatocytes or stellate cells in response to toxicants. It is also a mitogen involved in liver regeneration—proliferation of hepatocytes after partial hepatectomy [66]. For example, Norris et al. demonstrated that the levels of hepatic IL-6 in vitro and in vivo, after partial hepatectomy, were increased by exposure to hepatocyte growth factor (HGF) [66]. Stellate cells are an important source of HGF in the liver [37]. Thus, the higher levels of IL-6 observed in co-cultures may be the result of stellate cells signaling to hepatic cells via HGF or other morphogens. Another explanation is that stellate cells produce IL-6 directly and account for the difference in IL-6 levels between monocultures and co-cultures [67]. We therefore posit that in our case, IL-6 may function as a mitogen and an indicator of cell health. This would explain the declining levels of IL-6 after exposure to T-2 in co-cultures.

Similar to our study, Liu and colleagues reported that T-2 induced the expression of the pro-inflammatory cytokine TNF-α in BRL cells (a rat liver cell line) [68]. Additionally, TNF-α, IL-8, and IL-6 increased after T-2 exposure in PC12 cells used as neuron model cells [65]. Other authors reported that DON exposure to porcine hepatocytes, hepatocytes-Kupffer cell co-cultures, porcine alveolar macrophages, and U937 cells reduced IL-6 levels [59,69,70]. In addition, our observations are in line with those of Nagashima and colleagues that mycotoxins such as rubratoxin B do not induce MCP-1 secretion in HepG2 monocultures and lead to GM-CSF secretion in hepatic cultures [71].

Taken together, our results point to the expression of pro- (TNF-α, IL-8, GM-CSF, MCP-1) and anti-inflammatory cytokines (IL-1RA) in microfluidic hepatic spheroid cultures. These results also underscore that increasing culture complexity makes it more challenging to discern the cellular origins of signals and their mechanisms of action (e.g., discussion around IL-6). In the future, we are planning to explore the mechanism of activation of inflammatory cytokines. Nicotinamide N-methyltransferase (NNMT) is an enzyme present in the liver that regulates pro-inflammatory cytokines [72]. Xu et al. reported that NNMT may be a therapeutic target for DON-induced inflammation because NNMT overexpression decreased the pro-inflammatory cytokines [73,74]. Therefore, NNMT may be a target for attenuating liver inflammation after T-2 exposure.

## 4. Conclusions

In this work, we stablished and characterized microfluidic hepatic spheroid cultures comprised of HepG2 cells alone or in co-culture with stellate cells (LX2 cells). We used microfluidic cultures to evaluate exposure to mycotoxin T-2. We firstly demonstrated that hepatic spheroids formed faster and were more viable and functional in microfluidic devices than in commercial 3D cultures (Aggrewell plate). Then, we assessed the cytotoxicity, cell function, gene expression, and cytokine production of our hepatic cultures when exposed to the mycotoxin T-2. Importantly, the effects of the toxin were compared in microfluidic spheroid cultures of HepG2 cells alone and in co-cultures with stellate cells (LX2 cells).

We demonstrated that T-2 induced cell injury in 3D hepatic cultures, decreased albumin synthesis, and increased the production of inflammatory cytokines, including TNF-α. We also observed that the extent of injury was diminished by the presence of stellate cells in microfluidic spheroid cultures. The mechanisms by which stellate cells attenuate T-2 toxicity are multi-factorial and need further work. Our studies highlighted two potential roles. First, while the expression of metabolizing enzymes was similar between the two microfluidic culture formats, induction of these enzymes was significantly lower in microfluidic co-cultures compared to monocultures. This leads us to believe that hepatocytes in co-cultures may not experience the same levels of T-2. Whether this is due to T-2 partitioning into lipid droplets within stellate cells or whether transport of T-2 is inhibited in some way remains to be determined. Second, stellate cells may be the source of signals (e.g., IL-6) that promote hepatic phenotype and function, making hepatocytes less susceptible to insult with toxicants.

In summary, microfluidic 3D hepatic cultures were well-suited for testing T-2 mycotoxin. In the future, we will use this culture platform to evaluate therapeutic strategies for attenuating mycotoxin-induced inflammatory response. The microfluidic culture platform may be enhanced further by including more liver cell types and by using primary liver cells instead of cell lines.

## Figures and Tables

**Table 1:** Gene-specific primers for RT-PCR assays.

Gene Symbol	Gene Name	Tm (°C) Forward Primer/Revers Primer	Forward Primer/Revers Primer
GAPDH	Glyceraldehyde 3-phosphate dehydrogenase	57.2/57.7	TGTTGCCATCAATGACCCCTT/ CTCCACGACGTACTCAGCG
CYP1A1	Cytochrome P450 1A1	59.4/57.4	CATTAACATCGTCTTGGACC/ TCTTGGATCTTTCTCTGTACC
CYP1A2	Cytochrome P450 1A2	52.5/56.4	GCTTCTACATCCCCAAGAAAT/ ACCACTTGGCCAGGACT
CYP2E1	Cytochrome P450 2E1	57.5/57.8	GACACCATTTTCAGAGGATAC/ TTCATTCAGGAAGTGTTCTG
CYP3A4	Cytochrome P450 3A4	50.1/51.8	ACTGCCTTTTTTGGGAAATA/ GGCTGTTGACCATCATAAAAG
UGT1A1	UDP Glucuronosyltransferase 1A1	54.8/56.7	TTGATCCCAGTGGATGGC/ ATGCTCCGTCTCTGATGTACAAC
MDR1	Multidrug resistance mutation 1	56.3/56.1	GTCATCGCTGGTTTCGATGATG/ ATTTCCTGCTGTCTGCATTGTG
MDR2	Multidrug resistance protein 2	53.1/53.1	TCCAACTGTGCTTCAAGC/ GGCATCCACAGACATCAG

Tm: melting temperature.

## Data Availability

Data are contained within the article.

## Supplementary Materials

The following supporting information can be downloaded at: https://www.mdpi.com/article/10.3390/cells13110900/s1, Figure S1: Comparison of spheroid formation on Aggrewells vs. microfluidic devices. (A) Brightfield images. (B) Analysis of Aggrewell images. (C) Analysis of microfluidic images. (D) Metrics analyzed in both culture method for comparison.
