# Supplementary material for: Using Microfluidic Hepatic Spheroid Cultures to Assess Liver Toxicity of T-2 Mycotoxin
Source: Cells. 2024 May 24;13(11):900. doi: 10.3390/cells13110900 (PMC11172061; doi:10.3390/cells13110900)
Supplement: Supplementary file 1 [file cells-13-00900-s001.zip › cells-3008737-supplementary.pdf]

SUPPLEMENATRY INFORMATION

USING MICROFLUIDIC HEPATIC SPHEROID CULTURES TO ASSESS LIVER TOXICITY OF T-2 MYCOTOXIN

Mercedes Taroncher[1,2], Alan M. Gonzalez-Suarez[1], Kihak Gwon[1], Samuel Romero[3], Angel D. Reyes-Figueroa[3,4], Yelko Rodríguez-Carrasco[2], María-José Ruiz[2], Gulnaz Stybayeva[1], Alexander Revzin[1,±], Jose M. de Hoyos-Vega[1,±],

[1]*Department of Physiology and Biomedical Engineering, Mayo Clinic, Rochester, MN, 55901, USA*

[2]*Research Group in Alternative Methods for Determining Toxics Effects and Risk Assessment of Contaminants and Mixtures (RiskTox). Laboratory of Food Chemistry and Toxicology, Faculty of Pharmacy, University of Valencia, Av. Vicent Andrés Estellés s/n, 46100, Valencia, SPAIN*

[3]*Centro de Investigación en Matemáticas Unidad Monterrey, Apodaca, NL, 66628, MEXICO*

[4]*Consejo Nacional de Humanidades, Ciencias y Tecnologías, CDMX, 03940, MEXICO*

±Corresponding authors: dehoyos-vega.jose@mayo.edu; Revzin.alexander@mayo.edu

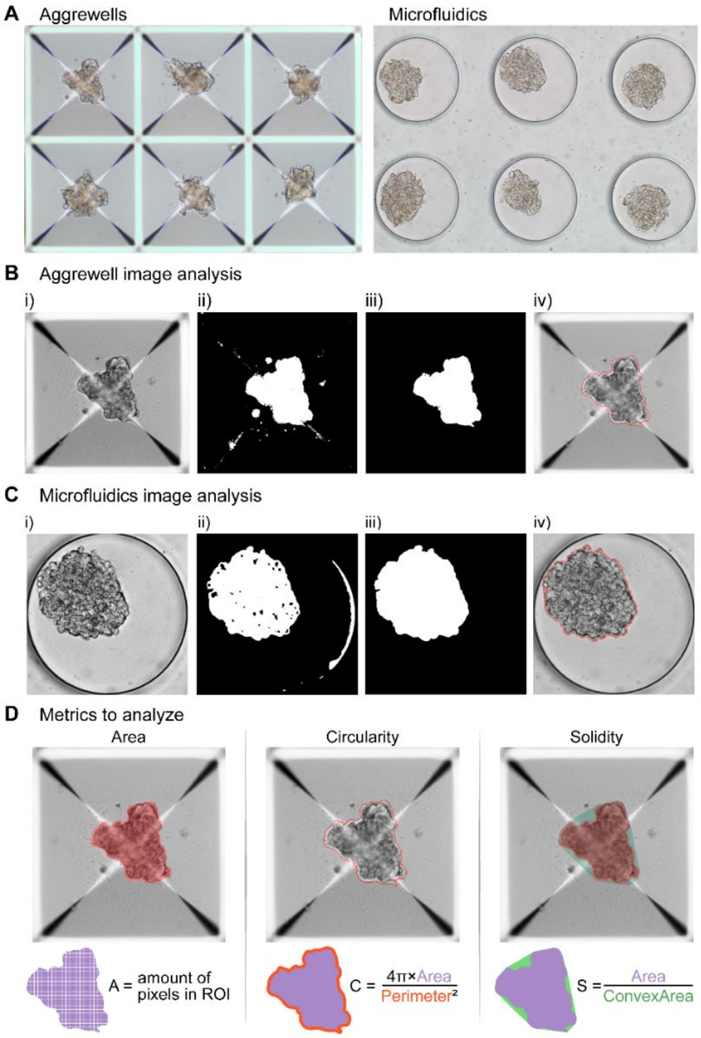

Figure S1. Comparison of spheroid formation on a microfluidic device and a commercial 3D plate. A) *Brightfield images.* ***B)*** *Analysis of images in an Aggrewell plate.* ***C)*** *Analysis of microfluidic images.* ***D)*** *Metrics analyzed in both culture method for comparison.*

Spheroid Formation Image Analysis

HepG2 cells were cultured in Aggrewell plate and in microfluidic devices as described in Methods section 2.3. Brightfield images were acquired just after cell seeding and then every 24 h for 5 days (**Fig. S1A**). Images were analyzed using Python and ImageJ for area, solidity, and circularity of each spheroid.

Image Analysis in Aggrewell Plate

Aggrewell images were segmented using Fiji (ImageJ) and the plugin Labkit [1] – Intuitive Pixel Classification [1]. The segmentation resulted in a binary image with the spheroid pixels localized (**Fig. S1Bii**), with some extra pixels. We then used Python (v3.11.5) with the OPENCV2 library (v4.8.1) to crop each well and maintain only the region of interest (largest element of the image),

followed by binary transformations to remove extra pixels, dilate thin lines, and fill the object area if any black spots were found. The area of the spheroid in pixel was determined as the area of the white element in the binary image (**Fig. S1Biii**). Finally, we determined the perimeter of the spheroid by defining the contour of the white object in the binary image. **Figure S1B** shows the segmentation steps for aggrewell images.

Image Analysis in a Microfluidic Device

To segment the spheroids in the microfluidic device images we only used Python and OPENCV2. The images from microfluidic devices show a smoother background compared to the aggrewell images, making the determination of the spheroid simpler. We first detected each circle in the image corresponding to the microwell walls, and deleted the pixels external to the circle. Then, we inverted the image color and applied a median blur filter to denoise the image and threshold to obtain the area of the spheroid as a binary image. We applied binary transformations to remove unwanted elements and filling black holes inside the spheroid to obtain the final spheroid area. Similarly to the aggrewell images, we determined the area and perimeter of the spheroid using the binary image. **Fig. S1C** shows the image segmentation steps for microfluidic cultures.

Metrics for Image Analysis

After image segmentation, we used two metrics to determine if the spheroid formation was different between aggrewell and microfluidic cultures: circularity and solidity [2,3]. For each spheroid at each time point, we calculated its circularity and solidity. The circularity of the spheroid was calculated by following equation (1) [2]. A value of 1 (100%) would mean that the spheroid shape is closer to a circle.

$$Circularity = \frac{4\pi \cdot Area}{Perimeter^2} \qquad (1)$$

For solidity, we use the spheroid perimeter and the determined a convex area (**Fig. S1D**) that represents a smooth spheroid. We then compare the area of the actual spheroid vs the convex area. This comparison returns a value between 0-1, where a value closer to 1 (100%) would mean that the spheroid has a smooth surface. After calculating the circularity and solidity of each spheroid, we plotted the results in scatter plots to show the differences between culture conditions.

References:

1. Arzt, M.; Deschamps, J.; Schmied, C.; Pietzsch, T.; Schmidt, D.; Tomancak, P.; Haase, R.; Jug, F. LABKIT: Labeling and Segmentation Toolkit for Big Image Data. *Front Comput Sci* **2022**, *4*, 10, doi:10.3389/fcomp.2022.777728.

2. Amaral, R.L.F.; Miranda, M.; Marcato, P.D.; Swiech, K. Comparative Analysis of 3D Bladder Tumor Spheroids Obtained by Forced Floating and Hanging Drop Methods for Drug Screening. *Front Physiol* **2017**, *8*, 284532, doi:10.3389/fphys.2017.00605.

3. Mora, C.F.; Kwan, A.K.H. *Sphericity, Shape Factor, and Convexity Measurement of Coarse Aggregate for Concrete Using Digital Image Processing*; **2000**, 30(3) 351-358, doi: 10.1016/S0008-8846(99)00259-8
